# Retinal Thickness Profiles in Parkinsonian Syndromes: Discerning Parkinson’s Disease, Multiple System Atrophy, and Progressive Supranuclear Palsy via Optical Coherence Tomography

**DOI:** 10.3390/biomedicines14010249

**Published:** 2026-01-22

**Authors:** Marko Svetel, Gorica Marić, Marija Božić, Tatjana Pekmezović, Igor Petrović, Jana Jakšić, Ana Dimitrijević, Una Lazić, Smiljana Kostić, Milica Knežević, Tiana Petrović, Sanja Petrović Pajić, Vesna Šobot, Jelena Vasilijević, Marina Svetel

**Affiliations:** 1 Clinic for Eye Diseases, University Clinical Center of Serbia, 11000 Belgrade, Serbia; marija.bozic@med.bg.ac.rs (M.B.); janajaksicmfub@gmail.com (J.J.); ana.m.dimitrijevic@gmail.com (A.D.); knezmima@gmail.com (M.K.); tianapetrovic93@gmail.com (T.P.); dr_sanja_petrovic@hotmail.com (S.P.P.); vesna.sobot01@gmail.com (V.Š.); bkjelena@gmail.com (J.V.); 2 Institute of Epidemiology, Faculty of Medicine, University of Belgrade, 11000 Belgrade, Serbia; gorica.maric@med.bg.ac.rs (G.M.); pekmezovic@orion.rs (T.P.); 3 Faculty of Medicine, University of Belgrade, 11000 Belgrade, Serbia; igor.n.petrovic@gmail.com (I.P.); marinasvetel@gmail.com (M.S.); 4 Clinic for Neurology, University Clinical Center of Serbia, 11000 Belgrade, Serbia; ulazic99@gmail.com; 5 Neurology Clinic, Military Medical Academy, 11000 Belgrade, Serbia; popovicsmiljana@gmail.com

**Keywords:** retinal thickness, Parkinson’s disease, atypical parkinsonism, multiple system atrophy, progressive supranuclear palsy, optical coherence tomography, biomarkers

## Abstract

**Background/Objectives**: Clinical differentiation between Parkinson’s disease (PD) and atypical parkinsonism (AP) remains complex. Current diagnostic procedures helpful in their distinction lack specificity, making non-invasive tools like optical coherence tomography (OCT) crucial in evaluating possible retinal changes as potential biomarkers. Our study examined the thickness of the ganglion cell inner plexiform layer complex (GCIPL), peripapillary retinal nerve fiber layer (RNFL) and macular segments in individuals with PD, multiple system atrophy (MSA), progressive supranuclear palsy (PSP), and healthy controls (HC). The objective of our study was to determine if OCT analyses can effectively discriminate PD patients from HC and whether retinal thickness can distinguish typical PD patients from those with AP. **Methods**: Research was an observational, cross-sectional study. Multiple retinal layers measured with OCT of PD and AP patients were compared with age- and sex-matched HC. An intergroup assessment was conducted. **Results**: Patients with PD and PSP exhibit a thinner GCIPL compared to HC, with no difference observed in the MSA group. GCIPL thickness between investigational groups does not differentiate between PD and AP. The RNFL and central macula thickness were statistically significantly reduced in all patient groups compared to HC. The RNFL was thinner in PSP compared to PD. Nearly all inner and outer macular segments were thinner in the investigational groups compared to HC. The preservation of outer nasal segments distinguished HC from both typical and AP. Patients with PSP and PD differed in the thickness of all macular segments, being thinner in PSP patients. **Conclusions**: Thickness of multiple retinal layers and macular regions might serve as a distinguishing feature between PD, AP and HC.

## 1. Introduction

Parkinson’s disease (PD) is a severe, progressive, neurodegenerative disorder that impacts a substantial number of individuals. According to a prediction, the global population of people with PD will rise by more than 12 million over the next 15 years [1]. Diagnosis of PD is clinical, and it relies on cardinal motor features such as tremor, bradykinesia, rigidity, and postural instability [2].

The main differential diagnostic dilemma is to separate PD from atypical parkinsonian syndromes, including multiple system atrophy (MSA) and progressive supranuclear palsy (PSP). Distinguishing clinically among them can be challenging, particularly in the early stages of the disease. These disorders have more complex clinical features which are characterized by the presence of autonomic disturbances, parkinsonism, and cerebellar symptoms in MSA [3,4], and postural instability, akinesia, cognitive disabilities, and gaze palsy in PSP [5].

In cases exhibiting fully manifested characteristic motor symptoms of PD, the error rates for clinical diagnosis may reach 24%, even within specialized institutions. Additionally, in clinicopathological series, the predominant misclassifications are MSA and PSP [6]. The diagnosis of MSA remains intriguing, especially during the initial consultation. The Parkinsonian variant of Multiple System Atrophy (MSA-P) can resemble PD. Clinicopathological research indicates that as many as 10% of individuals clinically diagnosed with PD may in fact have MSA, while up to 7% of those identified with MSA over their lifetime may have PD [7]. Furthermore, the clinical diagnosis of PSP was accurate in merely 25% of individuals during the initial visit, and in 63% by the last visit [8]. From a pathogenic perspective, PD and MSA are categorized as α-synucleinopathies, whereas PSP is defined as a 4-repeat tauopathy [9,10].

Searching for biomarkers, helpful for distinguishing typical and AP, is particularly important for clinicians. None of the currently available options are sufficiently specific for diagnosis, predicting future PD, assessing disease progression, or differentiating between PD and AP. Currently available modalities are costly (positron emission tomography and magnetic resonance imaging) or invasive (α-synuclein seed amplification test, skin biopsy) [11,12,13].

Visual impairments may serve as a significant potential marker linked to the diagnosis of PD, occurring in approximately 92% of PD patients [14]. While various visual symptoms emerge from impaired visual processing within the central nervous system, abnormalities at the ocular level, specifically in the retina, may also occur, and their contribution to visual disturbances should not be disregarded [15], rendering optical coherence tomography (OCT) studies a crucial non-invasive method for evaluating PD patients’ retinas.

OCT is an exceptional instrument for producing high-resolution images of retinal layers and their thickness, while simultaneously evaluating the integrity of retinal ganglion cells and their nerve fibers with high reproducibility [16,17].

Over a decade has elapsed since Inzelberg initially utilized OCT in patients with PD [18]. Further studies on the same topic showed conflicting results. Older studies claimed that there is no difference in thickness of retinal layers between PD patients and healthy controls (HC) [19,20,21], while recently published papers, mainly in the course of the last 5 years, showed that particular retinal structures were thinner in PD patients compared to HC [22,23,24,25,26,27,28,29]. Wagner et al. (2023) [30], in one of the most extensive and recent studies on the subject, also asserted that OCT analysis revealed significant thinning of the ganglion cell inner plexiform layer complex (GCIPL) and peripapillary retinal nerve fiber layer (RNFL) in PD patients compared to HC. In addition, in their prospective monitoring of a number of healthy individuals, among whom 0.1% developed PD, retinal layer thinning was detected even prior to the onset of motor symptoms [30].

To the best of our knowledge, studies that directly compare groups of patients with PD and AP are rare [31,32]. In one of these studies, Ma et al. (2023) suggested that OCT could be beneficial in the differential diagnosis of parkinsonian syndromes [32].

Our study was to examine the thickness of the GCIPL, RNFL, and macular segments in individuals with PD, MSA, PSP, and HC. The objective of our study was to determine if OCT analyses can effectively discriminate PD patients from HC and whether retinal thickness can distinguish typical PD patients from those with atypical presentations.

## 2. Materials and Methods

Our research was an observational, cross-sectional study. Patients were enrolled at the Department of Neurodegenerative Diseases at the Clinic for Neurology, University Clinical Centre of Serbia (UCCS), from 1 June 2022 to 31 December 2024, and thereafter referred to the Clinic of Ophthalmology, UCCS, for comprehensive ophthalmological assessment. The Ethics Committee of the Faculty of Medicine, University of Belgrade, approved our study (No. 25/III-18), as well as the Ethics Committee of UCCS (No. 1600/13). Informed consent was obtained from all participants.

### 2.1. Participants

The first group of patients consisted of 86 individuals (172 eyes) with PD. Patients underwent assessment by a neurologist specializing in movement disorders. All PD patients were diagnosed in accordance with the Movement Disorder Society (MDS) clinical diagnostic criteria for clinically confirmed PD [2].

The second cohort included 14 patients (28 eyes) diagnosed with PSP. They met the criteria established by the National Institute of Neurological Disorders and Stroke and the PSP Society (NINDS-SPSP) [5].

The third group of studied patients consisted of individuals with MSA, comprising eight patients (16 eyes). The diagnosis of clinically developed MSA was conducted in accordance with the MDS criteria for MSA diagnosis [3].

Healthy volunteers represented the control group. They did not have history of ocular issues. Only minor refractive errors, incipient cataract, and dry eye were allowed. Each investigational group had its own healthy control group matched by sex and age.

### 2.2. Clinical Assessment

Initially, clinicians and the study coordinator collected clinical and demographic information. The data included were current age, sex, age at disease onset and disease duration. The Hoehn–Yahr scale [33] and the MDS-Unified Parkinson’s Disease Rating Scale (MDS-UPDRS) were used to evaluate the severity of PD [34]. Prior to the assessment, subjects did not discontinue their dopaminergic medication. All patients and controls underwent a comprehensive ophthalmologic evaluation, which encompassed the assessment of best-corrected visual acuity (Snellen), measurement of intraocular pressure via applanation tonometry (Goldmann), slit-lamp biomicroscopy, gonioscopy, indirect ophthalmoscopy for fundus examination, and OCT.

### 2.3. Criteria for Exclusion

Patients were excluded from the study if they had a history of glaucoma or were glaucoma suspects, myopia exceeding −6 diopters, hyperopia of +6 diopters or greater, type 2 diabetes, or any other ocular or systemic conditions that could alter the retina and impact OCT analysis. Additionally, patients who underwent intraocular surgery or experienced trauma, as well as those with neurological diseases (multiple sclerosis or Alzheimer’s disease), were also excluded from the study. Patients who had severe gaze evoked palsy, blepharospasm, and apraxia of eyelid opening did not enter the investigational groups.

### 2.4. Optical Coherence Tomography Assessment

All patients and controls underwent examination with the SD-OCT device (RTVue-100, Optovue, Fremont, CA, USA) for measurement of the thickness, in micrometers, of the peripapillary RNFL as well as the outer and inner macular layers. In accordance with several other publications on this topic [22,32], results for each eye were acquired separately, and values recorded from each eye were included in the statistical analysis. An area of 4.9 mm centered on the optic disc was scanned for RNFL thickness assessment. A distinct scanning pattern, comprising 13 concentric circles and 12 radial lines, was utilized. The circles possessed diameters varying from 1.3 mm to 4.9 mm, with intervals of 0.3 mm. The scanning duration was 0.55 s, and 14,241 data points were used. The Early Treatment Diabetic Retinopathy Study (ETDRS) grid was employed to assess macular retinal thickness, segmented into nine divisions. The fovea had a center diameter of 1 mm, the parafovea ranged from 1 mm to 3 mm in diameter, and the perifovea extended from 3 mm to 5 mm in diameter. Furthermore, we divided the parafovea and perifovea into four quadrants: superior, inferior, nasal, and temporal. Criteria for data quality and inclusion: Only scans having a quality value of 40 or above were utilized in the subsequent study.

All OCT images were assessed by the same examiner. The primary independent variables included GCIPL thickness, average RNFL thickness, and total macular thickness assessed over nine segments (central macula, parafoveal, and perifoveal areas).

### 2.5. Statistical Evaluation

Data analysis employed descriptive and inferential statistics. Continuous variables were presented using mean and standard deviation, whereas categorical variables were presented using frequencies and percentages. To compare different characteristics between patients (PD, MSA, PSP) and healthy control participants, Student’s *t*-test and ANOVA were utilized in the case of continuous variables, and the Chi-square test was utilized for nominal data. All statistical analyses were conducted using SPSS (Statistical Package for Social Sciences), version 20. A *p*-value below 0.05 was considered significant. All *p*-values were presented with mean difference values and corresponding 95% Confidence Intervals (CI).

## 3. Results

### 3.1. Demographic Characteristics and Retinal Thickness Values of All Participants

Demographic characteristics of all participants are detailed in Table 1. There were no significant differences in age (*p* = 0.118) and sex ratio (*p* = 0.849) between PD, MSA, PSP, and controls. PD, MSA, and PSP patients differed in the mean disease duration (*p* = 0.010) as well as in age at disease onset (*p* = 0.004).

Our patients had a total UPDRS score of 40.7 ± 22.5, and the severity of their disease, assessed using the Hoehn and Yahr scale, was most often at stage II (40.7%), followed by I (23.3%) and III (15.2%).

Retinal thickness values for all patients’ groups are presented in Table 2.

### 3.2. Evaluation of Retinal Thickness of PD Patients and Healthy Control Subjects

**Chart 1:**
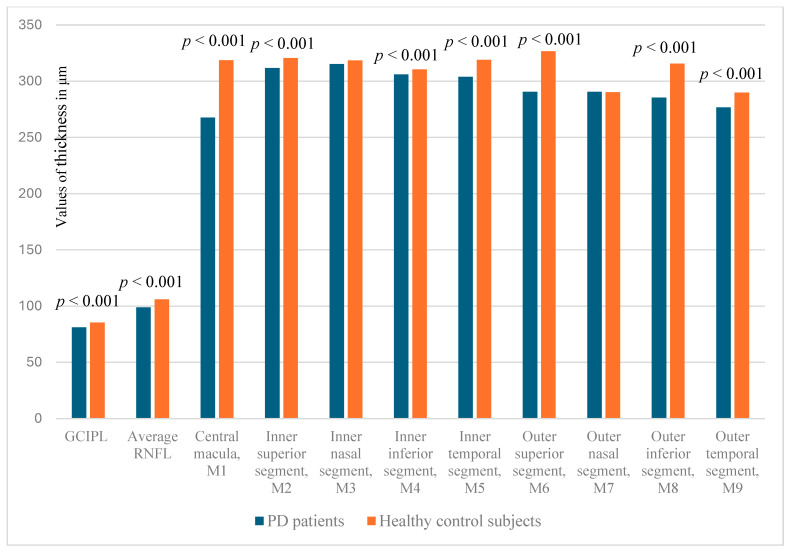
Thickness values for GCIPL, peripapillary RNFL, and macular segments (M1–M9) in PD patients compared to HC. GCIPL: ganglion cell/inner plexiform layer; RNFL: retinal nerve fiber layer; M1–M9: macular segments determined by the Early Treatment Diabetic Retinopathy Study (ETDRS) grid.

When we compared the thickness of the GCIPL, average peripapillary RNFL, and the macular thickness by segment in patients with PD and the HC group, it was shown that the GCILP and average RNFL were statistically significantly thinner in patients with PD compared to the HC group (*p* < 0.001; Mean Difference (MD): −4.379; 95% Confidence Interval (CI): −6.249/−2.509) and *p* < 0.001; MD: −7.164; CI: −9.069/−5.258, respectively) (Chart 1).

**Figure 1:**
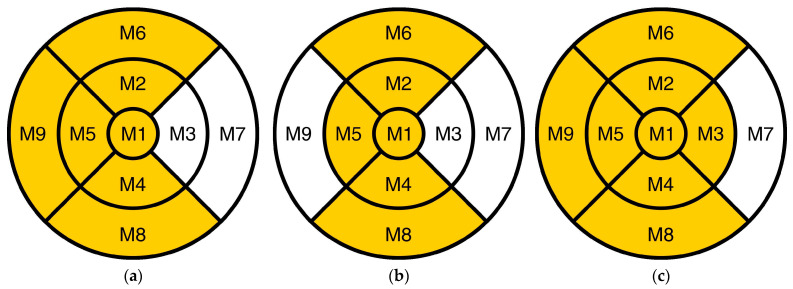
Comparison of macular retinal segment thickness between: (**a**) patients with PD and HC; (**b**) patients with MSA and HC; (**c**) patients with PSP and HC. Statistically significant differences in macular retinal segment thickness are colored in yellow.

Strong statistical significance was exhibited in all instances as follows: central macula (*p* < 0.001; MD: −50.953; CI: −56.418/−45.489), inner superior (*p* < 0.001; MD: −8.895; CI: −12.891/−4.899), inner inferior (*p* < 0.001; MD: −4.453; CI: −8.869/−0.038), inner temporal (*p* < 0.001; MD: −15.064; CI: −19.586/−10.542), outer superior (*p* < 0.001; MD: −36.134; CI: −40.093/−32.175), outer inferior (*p* < 0.001; MD: −30.099; CI: −34.911/−25.286), and outer temporal segment (*p* < 0.001; MD: −13.209; CI: −19.982/−6.436) (Chart 1, Figure 1a).

Therefore, PD patients have statistically significantly reduced retinal measures when compared to HC in GCIPL, RNFL, and all macular segments in terms of thickness, except for the inner and outer nasal segments.

### 3.3. Assessment of Retinal Thickness in MSA Patients and Healthy Control Subjects

**Chart 2:**
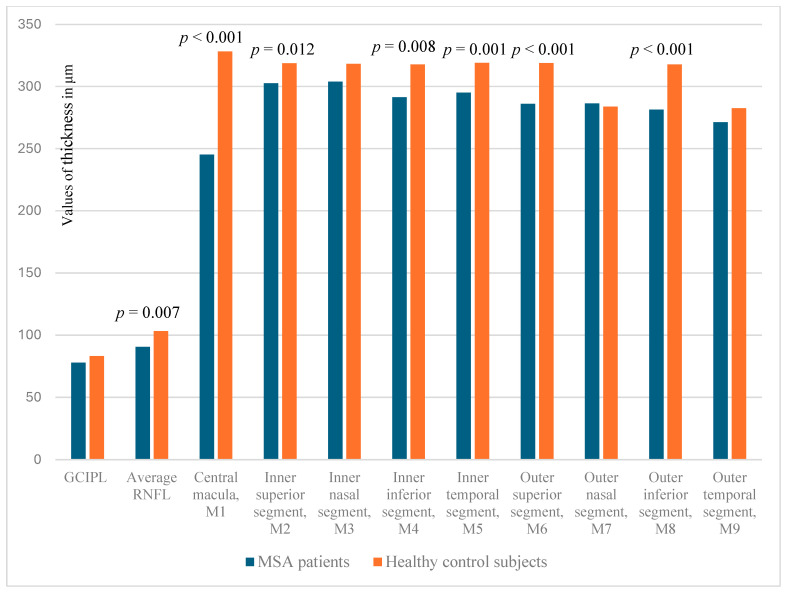
Thickness values for GCIPL, peripapillary RNFL, and macular segments (M1–M9) in MSA patients compared to HC. GCIPL: ganglion cell/inner plexiform layer; RNFL: retinal nerve fiber layer; M1–M9: macular segments determined by the Early Treatment Diabetic Retinopathy Study (ETDRS) grid.

Patients with MSA exhibited a statistically significant reduction in average RNFL thickness (*p* = 0.007; MD: −12.750; CI: −21.523/−3.977), whereas GCIPL thickness remained unchanged (Chart 2).

When it comes to macular segments, patients varied in the thickness of the central macula (*p* < 0.001; MD: −83.000; CI: −102.582/−63.418), inner superior (*p* = 0.012; MD: −16.250; CI: −28.619/−3.881), inner inferior (*p* = 0.008; MD: −26.313; CI: −45.180/−7.445), inner temporal (*p* = 0.001; MD: −24.063; CI: −37.066/−11.059), outer superior (*p* < 0.001; MD: −32.813; CI: −41.834/−23.791), and outer inferior segments (*p* < 0.001; MD: −36.375; CI: −49.655/−23.095) (Chart 2, Figure 1b).

Therefore, MSA patients have statistically significantly reduced retinal measures when compared to HC in RNFL and all macular segments in terms of thickness, except for the inner and outer nasal and outer temporal segments.

### 3.4. Analysis of Retinal Thickness in PSP Patients and Healthy Control Subjects

**Chart 3:**
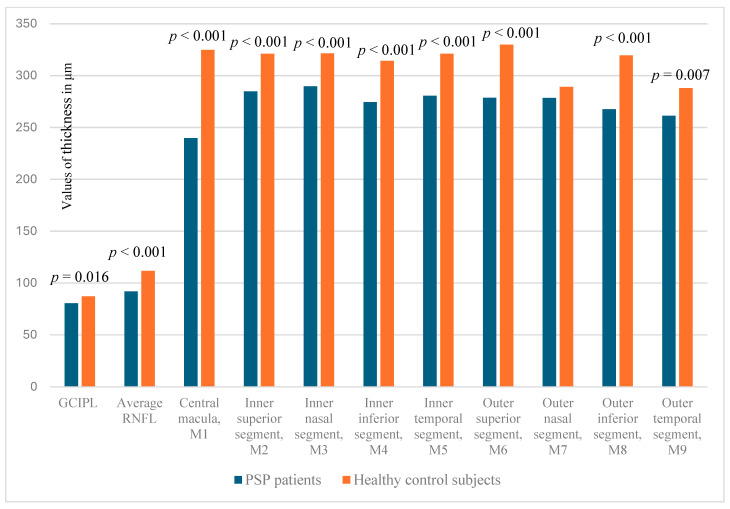
Thickness values for GCIPL, peripapillary RNFL, and macular segments (M1–M9) in PSP patients compared to HC. GCIPL: ganglion cell/inner plexiform layer; RNFL: retinal nerve fiber layer; M1–M9: macular segments determined by the Early Treatment Diabetic Retinopathy Study (ETDRS) grid.

Individuals diagnosed with PSP exhibit statistically significant reduction in GCIPL (*p* = 0.016; MD: −6.571; CI: −11.856/−1.287) and average RNFL thickness (*p* < 0.001; MD: −19.753; CI: −25.338/−14.167) compared to the HC group (Chart 3).

Patients with PSP differ in the thickness of macular segments from HC as follows: central macula (*p* < 0.001; MD: −85.000; CI: −98.961/−71.039), inner superior (*p* < 0.001; MD: −36.500; CI: −46.555/−26.445), inner nasal (*p* < 0.001; MD: −31.750; CI: −43.418/−20.082), inner inferior (*p* < 0.001; MD: −39.821; CI: −52.778/−26.865), inner temporal (*p* < 0.001; MD: −40.571; CI: −51.728/−29.415), outer superior (*p* < 0.001; MD: −51.286; CI: −62.387/−40.184), outer inferior (*p* < 0.001; MD: −51.893; CI: −65.260/−38.526), and outer temporal segment (*p* = 0.007; MD: −26.536; CI: −45.407/−7.665) (Chart 3, Figure 1c).

Therefore, PSP patients have statistically significantly reduced retinal measures when compared to HC in GCIPL, RNFL, and all macular segments in terms of thickness, except for the outer nasal segment.

### 3.5. Differences in Measured Retinal Parameters Among Patients with PD, MSA, and PSP

#### 3.5.1. Comparison of Patients with PD and MSA

Patients with PD did not differ from those with MSA in both GCIPL and average RNFL thickness (Table 3).

**Figure 2:**
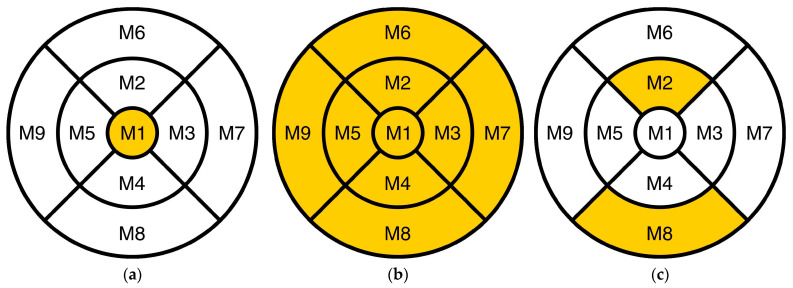
Comparison of macular retinal segment thickness between patients with (**a**) PD and MSA; (**b**) PD and PSP; (**c**) PSP and MSA. Statistically significant differences in macular retinal segment thickness are colored in yellow.

The thickness of the central macula was statistically significantly different (*p* = 0.009; MD: 22.464; CI: 5.624/39.303), while the thickness of all other macular segments did not differ (Figure 2a).

Therefore, PD and MSA patients differ only in central macula thickness, being thinner in MSA patients.

#### 3.5.2. Comparison of Patients with PD and PSP

In individuals with PSP compared to those with PD, there is no statistically significant difference in GCIPL; however, a significant difference is noted in average RNFL thickness (*p* = 0.002; MD: 6.986; CI: 2.706/11.266) (Table 3).

The thickness of the central macula (*p* = 0.000; MD: 27.901; CI: 14.799/41.004); all inner segments (superior (*p* = 0.000; MD: 26.924; CI: 17.371/36.476); nasal (*p* = 0.000; MD: 25.507; CI: 14.714/36.300); inferior (*p* = 0.000; MD: 31.606; CI: 19.373/43.840); and temporal (*p* = 0.000; MD: 23.419; CI: 13.398/33.439)), and all outer segments of the macula (superior (*p* = 0.015; MD: 11.980; CI: 2.321/21.640); nasal (*p* = 0.011; MD: 13.761; CI: 3.178/24.343); inferior (*p* = 0.003; MD: 17.886; CI: 6.336/29.437); and temporal (*p* = 0.008; MD: 13.498; CI: 3.593/23.402)) exhibited statistically significant differences (Figure 2b).

Therefore, PD and PSP patients differ in the thickness of the RNFL and all macular segments, being thinner in PSP patients.

#### 3.5.3. Comparison of Patients with MSA and PSP

Patients diagnosed with MSA did not differ significantly from those with PSP in terms of GCIPL and average RNFL thickness (Table 3).

When comparing macular segments, MSA patients differ from PSP in the thickness of the inner superior (*p* = 0.020; MD: 17.777; CI: 2.906/32.647) and outer inferior segment (*p* = 0.041; MD: 15.170; CI: 0.664/29.675) (Figure 2c).

Therefore, MSA and PSP patients differ only in the thickness of inner superior and outer inferior segments of the macula, being thinner in PSP patients.

## 4. Discussion

Despite inter-individual variability, our results show that in comparison with HC, all patients with typical and AP have a reduced RNFL and central macula thickness, while GCIPL was thinner only in PD and PSP patients. Nearly all inner and outer segments of the macula have reduced thickness in the investigational groups, but preservation of outer nasal segments distinguishes HC from both typical and AP. GCIPL thickness does not differentiate between patients with PD and AP, but the thickness of the RNFL might be helpful in differentiation of PD from PSP. In addition, patients with PD differ from those with PSP in the thickness of all macular segments.

The evidence from various studies suggests a definite involvement of the retina in parkinsonian disorders and robust correlations between retinal OCT dimensions and PD. In concordance with our findings, a number of authors observed that eyes of PD patients exhibited a notable decrease in average RNFL, GCIPL, and macular thickness [22,23,24,25,26,27,28,29]. Alterations in the central retina may improve the differentiation of patients with PD from healthy subjects, even during the onset or early stages of the disease. Wagner et al. (2023) recently demonstrated that a reduction in GCIPL and INL thickness occurs prior to the onset of motor symptoms [30]. Therefore, the authors revealed that thinner GCIPL correlated with an increased risk of PD development.

It was hypothesized that a thinning of RNFL, GCL, and IPL might be caused by different mechanisms. Evidence in the literature indicates that α-synuclein accumulates not only in PD brain tissue but also in the retina. The α-synuclein overexpression induces tyrosine hydroxylase amacrine cell neurodegeneration, which was shown in transgenic mice [35]. Atrophy of retinal ganglion cells as well as their nerve fibers could be a consequence of their reduced interaction with dopaminergic amacrine cells [22,25].

It was hypothesized that the thinning of the RNFL is the result of the loss of retinal ganglion cells and their axons [36]. However, other, mainly older, studies have suggested that patient and control groups cannot be differentiated by the thickness of RNFL [37,38]. Conversely, numerous investigations and meta-analyses determined that there was likely a significant decrease in average peripapillary RNFL thickness in PD [39,40,41] and yielded comparable findings consistent with our results.

As previously stated, the central macular segment (M1, foveal center with a diameter of 1 mm) in our study, which does not perfectly correspond to the foveal macula, was thinner in the PD cohort, like most other inner and outer macular segments. However, Sengupta et al. (2018) [36] revealed that the central foveal area was not reduced. The authors postulated that this finding is caused by foveal anatomy, with photoreceptors centrally located and the composite cellular network, comprising ganglion cells, localized peripherally [36]. Contrarily, but in accordance with our research, Bittersohl et al. (2015) demonstrated that the central macular area was significantly diminished in patients with PD and indicated that predominantly foveola displays delicate sensitivity and might be used as a potential biomarker [38]. The parafoveal and perifoveal macular segments were thinner in our sample, except for the inner and outer nasal segments. Analysis of the literature concerning macular segments yielded inconsistent results. Zhou, Tao and Li (2021), after examining 14 studies, demonstrated a significant decrease in the thickness of the macular fovea and all outer segments of the macula [22]. Additional studies verified that various segments exhibited reduced thickness, including the superior outer segment [42], the inner superior, and the outer temporal, nasal, and inferior segments [43], as well as the inner inferior, temporal, outer inferior, and foveal segments [44].

As retinal thinning was demonstrated in numerous studies in large cohorts of PD patients, these findings should not be neglected, although clinical application of these results is far away from the routine practice and recommendations.

In our cohort of patients with MSA, the average RNFL thickness was statistically considerably thinner, but GCIPL thickness did not differ from that of HC.

The preservation of GCIPL may be attributed to the localization of α-synuclein protein aggregation, characteristic for MSA. No aggregation of phosphorylated α-synuclein was observed in the ganglion cell layer [45], consistent with the neuropathological characteristic of α-synuclein deposition in oligodendrocytes in MSA [46]. Given the absence of oligodendroglial cells in the retina, aberrant α-synuclein may accumulate in oligodendroglial cells that myelinate axons and constitute a component of the optic nerve in MSA.

In our study, values for GCIPL thickness did not differentiate between PD and MSA groups. Mendoza-Santiesteban et al. (2015) showed that the GCIPL is thinner in PD compared to MSA patients [45]. Results were explained with the clinical characteristics indicating the absence of visual complaints in patients with MSA. The authors concluded that alterations in GCIPL were beneficial for distinguishing between MSA and PD. Our PD patients have a thinner GCIPL compared to MSA; nevertheless, this difference does not attain statistical significance.

As in our cohort, few studies presumed that the average RNFL thickness was thinner in MSA compared with that in HC, while other groups revealed that RNFL thickness in MSA was similar to that in HC and PD [47,48]. Our MSA patients have thinner RNFL in comparison with PD patients, but this difference also does not attain statistical significance.

We found that individuals with MSA exhibited differences in the thickness of all macular segments compared to HC, except for the inner and outer nasal segments and the outer temporal segment. The anatomy of retinal fibers indicates that thinner fibers in peripheral regions are anticipated due to the distribution of various types of retinal ganglion cells. P-cells possess smaller dendritic fields and are concentrated in the central retina, while M-cells possess broader retinal dendritic fields and are mostly situated in peripheral retinal areas. Oligodendroglial cells myelinate the axons of P-cells and M-cells after their departure from the eye [49]. M-cells necessitate increased myelination assistance from oligodendrocytes owing to their larger axonal diameter. In MSA, α-synuclein aggregates aberrantly in oligodendroglial cells, causing their degeneration [4], which results in a depletion of oligodendroglial support and exacerbates the degeneration of axons in M-cells.

In accordance, Mendoza et al. (2015) found a significant reduction in macular thickness only in the outer ring [45]. Conversely, as in our group, limited investigations suggested that the average central macular thickness was reduced in MSA compared to HC [47,48].

Our investigation indicated that there is no distinction in macular segment thickness between patients with PD and MSA. The sole recorded variation in our cohort was the thickness of the M1 segment, the central macular zone, which was thicker in individuals with PD. This is an unforeseen discovery, for which no elucidation has been identified in the literature. A group of authors discovered that the total thickness of the central retina remained largely normal in MSA [50] while drastically decreasing in PD, but in this report, patients were not compared directly.

Our PSP patients exhibit statistically significant differences from the HC group in GCIPL, average RNFL, and all macular segments in terms of thickness, except for the outer nasal segment. These findings align with the data from other studies [47,48].

In the literature, macular scans also revealed statistical discrepancies. In the study of Stemplewitz et al. (2017), the outer nasal sector of the PSP group was thicker than that of the control group [51].

In PSP, tau protein aggregates in various cell types, including neurons and glial cells [52]. The aggregation of tau protein may play a pivotal role in retinal degeneration in PSP. Tau deposits are present in the adult human retina [53].

Comparison between patients with PSP and PD reveals no statistically significant difference in GCIPL; however, differences are evident in the average thickness of RNFL and all macular segments. All referenced segments exhibited a statistically significant reduction in thickness among patients with PSP. Therefore, the thickness of the RNFL, as well as thickness of all macular segments, might be helpful in the differentiation of PD from PSP. Contrary to our findings, some studies showed a reduction in GCIPL thickness in PSP compared to that in PD [47,48]. Concerning RNFL, other research that analyzed retinal thickness reached identical conclusions [47,48,54].

In the meta-analysis conducted by Ma et al. (2022) [31], the authors incorporated 10 articles that met the inclusion criteria. The average RNFL and average central macular thickness were reduced in PSP and MSA compared to HC and were also diminished in PSP and MSA relative to PD [31].

Our patients diagnosed with MSA exhibited no significant differences from those with PSP regarding GCIPL thickness or average RNFL thickness.

We have to comment on the difference in disease duration between PD and AP patients. Our PD patients have a mean disease duration of ~7 years, whereas PSP and MSA patients have ~3 years. We are aware that disease duration might have a potential confounder effect on our results, influencing differences between our diagnostic groups. However, it is believed that disease duration of about 3 years is enough to see retinal changes in PSP patients [55]. Regarding the influence of disease duration on retinal thickness, results were conflicting. Wagner et al. (2023) revealed the presence of retinal changes even prior to the appearance of motor symptoms [30], and a number of other researchers detected that disease duration also does not influence retinal changes [51,56,57], while others claimed that the higher the severity of the disease (expectedly the longer the duration), the thinner the retina [26]. In our study, disease duration did not influence the retinal thickness in PD patients (unpublished data).

Study limitations. Comparing PD patients with AP is challenging due to the disparate and limited number of patients analyzed in studies concerning AP, attributable to the rarity of both conditions.

Various studies employed varied terminology to describe analogous or identical anatomical components.

The PD patients exhibited heterogeneity in age, disease duration, existing ocular conditions, treatment approaches, and cognitive status. Fortunately, in our sample, patients and HC were matched for age and sex.

Heterogeneity in disease duration exists between PD and AP groups due to the severity and rapid clinical course of AP rendering patients mostly immobile and incapable of participating in the study. It is not possible to negate the influence of the disease duration as a confounder in the interpretation of our results.

Also, when performing analyses, we included measurements from both eyes of the same individual as independent observations, i.e., we did not perform intra-subject correlation. Therefore, variance in this case might have been underestimated, and the risk of type I error increased.

The discrepancies observed between PD clinical ratings and various retinal measurements can be attributed to the distinct OCT devices employed, their hardware, the diverse procedures utilized for retinal studies, and the different stages of the illness present in individuals at the time of examination. Results acquired from different OCT devices are not comparable and cannot be used interchangeably.

It is important to emphasize that the raw *p*-values in our multiple simultaneous comparisons of OCT parameters were not corrected, and thus, it might have increased the probability of type I error, i.e., probability of significant findings. Therefore, our conclusions, especially those with borderline significance, should be interpreted with caution.

Another limitation of our study refers to the absence of data on the sensitivity and specificity of the used tests. However, we included some measures of effect size (mean difference and corresponding 95% Confidence Intervals).

## 5. Conclusions

Our results show that in comparison with HC, all patients with typical and AP have a reduced RNFL and central macula thickness, while GCIPL was thinner only in PD and PSP patients.

Nearly all inner and outer segments of the macula have reduced thickness in the investigational groups, but preservation of outer nasal segments distinguishes HC from both typical and AP.

GCIPL thickness does not differentiate between patients with PD and AP, but the thickness of the RNFL might be helpful in differentiation of PD from PSP.

In addition, patients with PD differ from those with PSP in the thickness of all macular segments.

The inconsistency and irreproducibility of results regarding GCIPL, RNFL, and macular segment thickness across numerous studies indicate that the potential of OCT retinal imaging as a biomarker for diagnosis and differential diagnosis of PD and AP requires additional investigation. However, a number of recently published studies testing large cohorts of patients which consistently revealed retinal thinning in PD and AP pointed out that these results should not be overlooked despite the fact that at the moment, they are not consistent enough to be accepted as biomarkers.

## Figures and Tables

**Table 1:** Demographic and clinical characteristics of patients with PD, MSA, and PSP.

	PD	MSA	PSP
Gender (m:f)	49:37 (57:43%)	6:2 (75:25%)	9:5 (64:36%)
Actual age ∞	59.2 ± 11.9	61.1 ± 4.9	65.7 ± 5.0
Age at disease onset ∞	52.1 ± 12.6	58.4 ± 5.2	62.8 ± 4.6
Disease duration ∞	6.7 ± 5.6	2.8 ± 1.2	2.9 ± 1.5

PD: Parkinson’s disease; MSA: multiple system atrophy; PSP: progressive supranuclear palsy; ∞—data are presented in age with mean value and SD.

**Table 2:** Retinal thickness values in patients with PD, MSA, and PSP.

	PD Patients	MSA Patients	PSP Patients
GCIPL	81.1 ± 7.2	77.9 ± 7.5	80.5 ± 10.9
Average RNFL	98.9 ± 10.0	90.5 ± 16.1	91.9 ± 13.2
Central macula, M1	267.7 ± 32.4	245.2 ± 35.4	239.8 ± 33.8
Inner superior segment, M2	311.7 ± 23.5	302.6 ± 19.9	284.8 ± 25.3
Inner nasal segment, M3	315.2 ± 26.7	303.9 ± 27.9	289.7 ± 27.9
Inner inferior segment, M4	306.0 ± 22.1	291.4 ± 32.8	274.4 ± 30.5
Inner temporal segment, M5	303.9 ± 24.8	295.1 ± 21.8	280.5 ± 26.0
Outer superior segment, M6	290.6 ± 23.6	286.1 ± 15.4	278.6 ± 26.5
Outer nasal segment, M7	290.5 ± 25.2	286.4 ± 16.5	278.5 ± 23.3
Outer inferior segment, M8	285.5 ± 28.2	281.4 ± 23.6	267.6 ± 31.9
Outer temporal segment, M9	276.7 ± 26.9	271.3 ± 23.8	261.4 ± 27.0

PD: Parkinson’s disease; MSA: multiple system atrophy; PSP: progressive supranuclear palsy; GCIPL: ganglion cell/inner plexiform layer; RNFL: retinal nerve fiber layer; M1–M9: macular segments determined by the Early Treatment Diabetic Retinopathy Study (ETDRS) grid.

**Table 3:** Differences in GCIPL and RNFL thickness among patient groups.

	PD and MSA	PD and PSP	MSA and PSP
GCIPL	0.157	0.835	0.480
Average RNFL	0.056	0.002 **	0.754

PD: Parkinson’s disease; MSA: multiple system atrophy; PSP: progressive supranuclear palsy; GCIPL: ganglion cell/inner plexiform layer; RNFL: retinal nerve fiber layer; ** *p* < 0.01.

## Data Availability

The data supporting this study’s findings are available from the corresponding author upon reasonable request.

## Abbreviations

The following abbreviations are used in this manuscript:

PD	Parkinson’s disease
MSA	Multiple system atrophy
PSP	Progressive supranuclear palsy
OCT	Optical coherence tomography
RNFL	Retinal nerve fiber layer
GCIPL	Ganglion cell/inner plexiform layer
UCCS	University Clinical Center of Serbia
HC	Healthy controls
MDS	Movement Disorder Society
MDS-UPDRS	MDS-Unified Parkinson’s disease rating scale
ETDRS	Early Treatment of Diabetic Retinopathy Study
SPSS	Statistical Package for Social Sciences
M1	Central macula
M2	Inner superior segment
M3	Inner nasal segment
M4	Inner inferior segment
M5	Inner temporal segment
M6	Outer superior segment
M7	Outer nasal segment
M8	Outer inferior segment
M9	Outer temporal segment
